# Comparative study of oral versus parenteral crocin in mitigating acrolein-induced lung injury in albino rats

**DOI:** 10.1038/s41598-022-14252-4

**Published:** 2022-06-17

**Authors:** Walaa Abdelhaliem Rashad, Samar Sakr, Ayat M. Domouky

**Affiliations:** 1 Human Anatomy and Embryology Department, Faculty of Medicine, Zagazig University, Alsharquiah, Egypt; 2 Forensic Medicine and Clinical Toxicology Department, Faculty of Medicine, Zagazig University, Alsharquiah, Egypt

**Keywords:** Cell biology, Environmental sciences, Anatomy, Medical research

## Abstract

Acrolein (Ac) is the second most commonly inhaled toxin, produced in smoke of fires, tobacco smoke, overheated oils, and fried foods; and usually associated with lung toxicity. Crocin (Cr) is a natural carotenoid with a direct antioxidant capacity. Yet, oral administration of crocin as a natural rout is doubtful, because of poor absorbability. Therefore, the current study aimed to compare the potential protective effect of oral versus intraperitoneal (ip) crocin in mitigating Ac-induced lung toxicity. 50 Adult rats were randomly divided into 5 equal groups; Control (oral-saline and ip-saline) group, Cr (oral-Cr and ip-Cr) group, Ac group, oral-Cr/Ac group, and ip-Cr/Ac group; for biochemical, histopathological, and immunohistochemical investigations. Results indicated increased oxidative stress and inflammatory biomarkers in lungs of Ac-treated group. Histopathological and immunohistochemical examinations revealed lung edema, infiltration, fibrosis, and altered expression of apoptotic and anti-apoptotic markers. Compared to oral-Cr/Ac group, the ip-Cr/Ac group demonstrated remarkable improvement in the oxidative, inflammatory, and apoptotic biomarkers, as well as the histopathological alterations. In conclusion, intraperitoneal crocin exerts a more protective effect on acrolein-induced lung toxicity than the orally administered crocin.

## Introduction

Acrolein (Ac) represents the second most commonly inhaled toxin in smoke of fires, after carbon monoxide^1^. It is a product of incomplete combustions (e.g., gasoline and plastic materials), smoking of tobacco products, and overheated oils; mostly through lipid peroxidation procedures^2,3^. Ac was listed by the United States Environmental Protection Agency (USEPA) as a high priority toxic chemical. Inhalation of Ac results in nasal irritation, bronchial hyperactivity, mucus production, and has been linked to pulmonary edema and chronic obstructive pulmonary disease (COPD)^4,5^ Ac metabolism necessitates reduced glutathione (GSH) conjugation^6^. If Ac is not totally conjugated and fully eliminated, the cellular redox balance will be markedly disturbed by GSH consumption^7^ and decreased antioxidant enzymes e.g., superoxide dismutase (SOD), and GSH peroxidase (GPx)^8^.

Crocin (Cr) is the main active carotenoid of Crocus sativus linne (saffron) acting as a biological antioxidant. Cr demonstrated protective effects as a naturally derived antioxidant in vitro and in vivo models of toxicity^9,10^. In pharmacological studies, Cr has revealed anti-inflammatory, anticonvulsant, and anti-tumor activities^11^. Moreover, Cr has shown protective effects against chemotherapeutic agents and genotoxins-induced oxidative stress in albino mice^12^. Beside the direct antioxidant activity, Cr has improved the anti-inflammatory capacity of the lung as proved by Dianat et al.^13^. However, Xi et al.^14^ and Lautenschläger et al.^15^ have reported the presence of trace amounts of Cr in systemic circulation after oral administration. Accordingly, the efficacy of oral Cr seems to be limited. Therefore, the aim of the present study was to compare the potential protective effect of oral versus (vs) intraperitoneal Cr on Ac-induced oxidative, inflammatory, apoptotic, and histopathological alterations in lungs of adult male albino rats. Up to our knowledge, this study is the first to evaluates the protective effect of oral versus intraperitoneal crocin on Ac-induced lung toxicity.

## Material and methods

### Chemicals

Crocin (C_44_H_64_O_24_) in the form of deep red colored powder, was acquired from Sigma-Aldrich, St. Louis, MO, USA (CAS number 42553-65-1). Acrolein (C3H4O) in the form of colorless to yellowish flammable liquid at room temperature with unpleasant odor, high vapor pressure (274 mm Hg @ 25 °C), water solubility (208 g/L @ 20 °C), molecular weight (56.06 g/mol), and specific gravity (0.8389 @ 20 °C)^16^; was purchased from El-Gomhouria Company for pharmaceutical, Egypt. All chemicals used in current study were of the highest commercially available quality.

### Animals

All experimental procedures were carried out in conformity with the appropriate standards and regulations of the Institutional Animal Care and Use Committee, Zagazig University (ZU-IACUC), approval number (*ZU-IACUC/3/F/189/2021*) and conducted in accordance with ARRIVE guidelines. 50 adult male albino rats weighing 150–200 gm were used in this study, divided into 5 groups (10 rats each). Rats were obtained from the animal house, Faculty of Medicine, Zagazig University. The rats were kept in separate cages (2 rats per cage) and fed normal rat chow under regular laboratory and ambient conditions. The animals were handled according to the guidelines provided out in the National Institute of Health’s Guide for the Care and Use of Laboratory Animals.

### Experiment protocol

50 Adult rats were randomly divided into 5 equal groups (10 rats each); Control (oral-saline & ip-saline) group, Cr (oral-Cr & ip-Cr) group, Ac group, oral-Cr/Ac group, and ip-Cr/Ac group. Control group was sub-grouped into two groups; (oral-saline): 5 rats received oral 0.5 cm physiological saline/daily for 2 weeks, and (ip-saline): 5 rats received ip 0.5 cm physiological saline/daily for 2 weeks; Cr group was sub-grouped into two groups (oral-Cr): 5 rats received oral 60 mg/kg crocin daily for 2 weeks^17,18^, and (ip-Cr ): 5 rats received ip 50 mg/kg crocin once daily for 2 weeks^13,19^; Ac group was subjected to acute acrolein inhalation (10 ppm for 12 h, daily for 2 weeks) in a closed inhalation chamber in accordance with the previously published method^20^; oral-Cr/Ac Group received oral 60 mg/kg crocin once daily one hour before acrolein inhalation as described; and ip-Cr/Ac group received ip 50 mg/kg crocin one hour before acrolein inhalation as described.

### Inhalation methodology of acrolein

Nitrogen gas (3–15 ml/min) was passed over a 3-ml reservoir of liquid acrolein to produce acrolein vapor. This combination was diluted in a 0.32 m^3^ stainless steel chamber with high efficiency particle filtered room air (400 ml/min). The concentrations of acrolein in samples collected with a succession of two glass fritted impingers containing 10 ml of 96 percent ethanol; were determined. A portion of these samples was combined with 50 mg of hexylresorcinol (Sigma, St. Louis, MO), 2.1 mg of mercury chloride (Aldrich, Milwaukee, WI), and 29.7 mg of trichloroacetic acid (Aldrich, Milwaukee, WI) (Fisher, Fair Lawn, NJ). In equal quantities, samples and known standards were heated (65 °C, 15 min) and cooled (22 °C, 15 min), and the absorbance at 605 nm was measured with a spectrophotometer (Beckman DU-64).

### Samples preparation

Twelve hours after the last treatments, rats were weighed, anaesthetized using 50 mg/i.p sodium phenobarbital, and then sacrificed. The trachea of each rat was cannulated and chest cavity was opened by a midline incision. The right lungs were weighed, then ligation of left main stem bronchus was done, and lavage of the right lung was undertaken using 3 mL of 0.9% saline through the cannula (24G size and 16 mL/min flow rate). This was repeated for 3 times with application of a gentle pressure to the thorax to magnify retrieval volume. Fluid recovery was kept above 80% for all rats. Then, right lungs were utilized to measure dry weight.

To eliminate all red blood cells and clots, tissues were extensively perfused with a 50 mM sodium phosphate buffer saline (100 mM Na2HPO4/NaH2PO4) (pH 7.4) and 0.1 m ethylenediaminetetraacetic acid (EDTA) solution prior to dissection. Then, the harvested left lung of each rat was divided into three parts: a small part of the upper lobe for DNA extraction, the remaining part of upper lobe for homogenate tissue analyses, and the lower lobe for the histopathological examinations.

### Anthropometric measures

Measuring body weight: each animal was placed in sealed plastic container and weighed day before the experiment and on the last day. The results were written in a record for each labeled rat. Moreover, lung wet-to-dry weight (W/D) ratio was measured, right lungs were excised, each lung was weighed (wet lung weight), and then placed in an oven at 80 °C for 48 h to obtain the dry weight. To assess tissue edema, the wet lung to dry lung ratio was measured. Lung index was also determined using the formula “Lung index = wet lung weight /body weight × 1000” as described by Mehrabani et al.^21^.

### Broncho-alveolar lavage examination

The obtained broncho-Alveolar Lavage (BALF) was centrifuged (2000 × g for at 4 °C for10 min). Then, the supernatant was aliquoted and stored at − 80 °C for assay of tumor necrosis factor (TNF)-α, interleukin (IL)-6, and macrophage inflammatory protein (MIP)-2. Analyses were performed according to the manufacturer's protocol using commercially available enzyme-linked immunosorbent assay (ELISA) kits of Abcam (TNF-α: ab46070; IL-6: ab100772, UK) and MIP-2 ELISA kits of Biosource International, Camarillo, USA. Results were expressed as pg/mL.

### Homogenate tissue analysis for oxidative stress parameters

Tissues were homogenized in ice-cold phosphate buffers (50 mM, pH 7.4) 5 times as their tissue weight and centrifuged at 5000 rpm for 30 min. Then, supernatants were preserved in a deep freeze until being used for the following assays:

#### Reactive oxygen species (ROS)

Concentration of ROS in the supernatant of tissue homogenate was determined using ROS ELISA kits (Cat. No. MAB7475; R&D Systems, Inc., Minneapolis, MN, USA) as described by the manufacturer. Using a Tecan Sunrise microplate reader (Tecan Group, Ltd., Männedorf, Switzerland), the absorbance was determined spectrophotometrically at 450 nm. Results were expressed as U/mg protein.

#### Malondialdehyde (MDA)

Lipid peroxidation product was determined using kits of Biodiagnostic (CAT. No. MD2529, Giza, Egypt). Absorbance of pink-colored complex of thiobarbituric acid and MDA was measured spectrophotometrically at 532 nm. Results were expressed as nmol/mg.

#### Reduced glutathione (GSH)

Reduced glutathione (GSH) was determined using kits of MyoBiosource (Cat. No. MBS265966, San Diego, USA). Ellman’s reagent was used. The absorbance was measured at 412 nm and expressed as nmol/mg.

#### Protein carbonyls (PCOs)

The formed PCOs were measured using MyBiosource kits (Cat. No. MBS2600784, San Diego, USA). Principle relied on the derivatization of carbonyl group with 2,4-dinitrophenylhydrazine (DNPH) forming the stable 2,4-dinitrophenylhydrazone (DNP)-carbonyl adduct which was quantified spectrophotometrically at 365 nm. The results were expressed as nmoles of protein carbonyls/mg proteins.

#### 8-hydroxydeoxy guanosine levels (8-OHdG)

Extraction of genomic DNA was undertaken from a small piece of left lung using a Qiagen DNeasy blood and tissue kit (Qiagen, Germany). Then, DNA extract was digested by DNase I [Takara Biotechnology (Dalian) Co., Japan], and Nuclease P1 as described by the manufacturer. Thereafter, digested DNA was incubated at 37 °C for 30 min with alkaline phosphatase at 1 unit AP/100 µg DNA, then, boiled for 10 min, and finally placed on ice. The 8-OHdG level was determined using 8-OHdG ELISA kits obtained from Abcam (CAT. No. ab201734, Tokyo, Japan). Results were expressed as ng/mL lung extract.

### Histopathological examination for acrolein-induced lung pathology

The lung tissue samples were fixed in a 10% neutral buffered formalin solution for general histology. Then, processed for paraffin wax embedding, sectioned at 5 mm thickness, and stained with hematoxylin and eosin (H&E) and Masson’s trichrome. 5 different non-overlapped sections from each animal, were examined and evaluated under light microscope (Anatomy Department, Zagazig University) and photographed.

### Immunohistochemical examination for apoptotic markers

The streptavidin–biotin immunoperoxidase method was used for immunohistochemistry. Formalin-fixed, paraffin-embedded blocks were mounted on positively charged glass slides, deparaffinized in xylene, and rehydrated in graded alcohol to produce 3–5 m thick sections. In citrate buffer at PH 6, heat induced antigen retrieval was done for 20 min. For 10 min, sections were treated with 3% hydrogen peroxide to inhibit endogenous peroxidase activity, then rinsed in phosphate buffered saline (PBS). The slides were then incubated overnight with primary antibodies: (anti-BAX (Abnova™ 1:100 dilution, ab 16152320, Lab Vision, California USA) and anti-Bcl2 (ready to use (ab16896274), Lab Vision, California USA). Incubation with a secondary antibody and product visualization were performed with diaminobenzidine chromogen. Counterstaining with Mayer’s hematoxylin and slides washing with distilled water and PBS were done. PBS was used instead of primary antibody as negative controls. 10 fields from 3 sections from each rat were coded enabling blind examination and evaluation under light microscope (Anatomy Department, Zagazig University) and photographed.

### Image analysis

10 fields from 5 sections for each rat in each group were coded enabling blind examination and evaluation, assessment of images was analyzed using imageJ software (ImageJ/Fiji 1.46r, https://imagej.nih.gov/ij/index.html). Regarding to × 400 H&E obtained fields. Interstitial thickening, inflammatory cell infiltration, congestion, and edema were all classified as 0 (absent), 1 (weak), 2 (moderate), 3 (strong), or 4 (severe) for grading of lung injury. The total lung injury score was calculated by adding up the individual scores of each category^22–24^. Moreover, Alveolization was estimated at 200 magnification fields by the radial alveolar count (RAC), alveolar number/field, and mean linear intercept (MLI) was calculated by dividing the total length of a line (in micrometers) drawn across the entire field by the total number of alveolar intercepts encountered along the length of the line^25^. In addition, in Masson's trichrome obtained fields, Lung fibrotic score was graded from 0 (normal) to 4 (severe) and the intensity of immunolabelling with the anti- BAX and anti-Bcl2 antibodies for alveolar cells in lung tissues in different studied groups were scored as 0 (normal) to 4 (severe). All parameters were assessed blindly by two different pathologists and the average was taken.

### Statistical analysis

The collated biochemical and morphometric data were statistically analyzed using the SPSS program (Statistical Package for Social Science) version 18.0. The normal distribution of the data was assured using the Shapiro–Wilk test, where the *p* value was found to be greater than 5%. For ease of presentation, normally distributed data were summarized by mean and standard deviation. As a key assumption of the One Way ANOVA, the researchers assessed homogeneity of variance among the compared groups using Levene's Test for equality of variances, where the *p* value was estimated to be more than 0.05. Consequently, the One Way ANOVA was conducted to test the significance of the difference in the mean of the compared groups. Multiple comparisons were estimated by Tukey HSD post-hoc test. With a 95% confidence level, a value of *p* < 0.05 was statistically significant.

### Ethics approval

All experimental procedures were carried out in conformity with the appropriate standards and regulations of the Institutional Animal Care and Use Committee, Zagazig University (ZU-IACUC/3/F/189/2021).

## Results

### Comparing control and crocin subgroups

Oral and ip rats in the control and crocin subgroups showed no significant difference (*p* > 0.05) when compared to each other regarding all the anthropometric, biochemical and morphometrical parameters. Consequently, oral-saline group was used for statistical comparison with other groups all through the study.

### Effect of acrolein ± crocin on anthropometric measures

When compared to the oral-saline group, acrolein treatment in Ac group resulted in a significant (*p* < 0.05) decrease in body weight, and a significant elevation in wet lung weight (*p* < 0.05) and lung index (*p* < 0.001) with no significant difference in dry lung weight. Moreover, wet/dry lung weight (W/D%) was significantly increased (p˂0.001) indicating lung edema.

Upon combination with oral-Cr, body weight, wet and dry lung weight showed no significant difference versus oral-saline and Ac groups. While lung index remained significant increase (* p* < 0.05) versus oral-saline group, but W/D % showed significant decrease (*p* < 0.05) versus Ac group. Likewise, ip-Cr/Ac group demonstrated no significant difference in wet and dry lung weight versus oral-saline nor Ac groups. While body weight demonstrated significant increase (*p* < 0.05), lung index demonstrated significant decrease (*p* < 0.05), and W/D % showed significant decrease (*p* < 0.001) versus Ac group. Comparing oral and intraperitoneal Cr/Ac groups showed no significant difference regarding all anthropometric measures (Table 1).

**Table 1:** Effect of crocin ± acrolein on anthropometric parameters in different studied groups.

	Control group		Cr group		Ac group (n10)	Oral-Cr/Ac group (n10)	ip-Cr/Ac group (n10)	*p* value
	Oral-saline (n5)	ip-saline (n5)	Oral-Cr (n5)	ip-Cr (n5)				
Body weight (gm)	242.6 ± 21.8	232.6 ± 23.6	233.5 ± 14.7	229.2 ± 16.4	201.3 ± 11.3^a^	221.4 ± 24.5	232.5 ± 18.2^c^	0.0031
Wet lung weight (gm)	1.4 ± 0.2	1.5 ± 0.4	1.6 ± 0.4	1.4 ± 0.6	2.3 ± 0.7^a^	1.9 ± 0.6	1.8 ± 0.4	0.0183
Lung index*	6.4 ± 2.1	6.5 ± 1.3	7.2 ± 3.1	7.9 ± 3.5	10.2 ± 0.7^a^	8.1 ± 1.3^c^	6.9 ± 0.9^c^	0.0013
Dry lung weight (gm)	0.52 ± 0.14	0.49 ± 0.1	0.5 ± 0.04	0.53 ± 0.05	0.6 ± 0.2	0.56 ± 0.6	0.62 ± 0.7	0.9966
W/D %	3.3 ± 1.1	2.9 ± 1.5	3.2 ± 0.7	3 ± 0.4	6.4 ± 1.7^b^	4.7 ± 1.3^c^	3.9 ± 0.6^d^	< 0.001

One-way ANOVA, and Tukey HSD Post-hoc Test, *p* > 0.05: no significant differences, *p* < 0.05: significant differences.

*Lung index = wet lung weight (gr)/body weight (gr) × 1000; wet/ dry lung weight%.

^a^*p* < 0.05 versus oral-saline group.

^b^*p* < 0.001 significant versus oral-saline group.

^c^*p* < 0.05 versus Ac group.

^d^*p* < 0.001 significant versus Ac group.

^e^*p* < 0.05 versus oral-Cr/Ac group.

^f^*p* < 0.001 significant versus oral-Cr/Ac group.

### Effect of acrolein ± crocin on oxidative stress parameters

Acrolein treatment resulted in a significant elevation (*p* < 0.001) in levels of oxidative stress parameters (ROS, MDA, PCO, and 8-OHdG) and decrease in GSH levels in Ac-treated group when compared to the oral-saline group. Upon combination with oral-Cr, results demonstrated a significant reduction (*p* < 0.05) in oxidative stress parameters and a significant increase (*p* < 0.05) in GSH levels in oral-Cr/Ac group compared to Ac-treated group. Meanwhile, a significant increase (*p* < 0.001) in oxidative stress indices and decrease in GSH levels was still detected in oral-Cr/Ac group in comparison with the oral-saline group. On the other hand, ip-Cr/Ac group demonstrated a significant reduction (*p* < 0.001) in ROS, MDA, PCO, and 8-OHdG levels, as well as a significant elevation (*p* < 0.001) in GSH levels when compared to Ac-treated group, and a significant (*p* < 0.05) difference when compared to oral-saline group. The comparison between the oral-Cr/Ac group and ip-Cr/Ac group revealed a significant reduction (*p* < 0.001) in ROS, MDA, PCO, and 8-OHdG, and a significant elevation (*p* < 0.001) in GSH levels in ip-Cr/Ac group compared to oral-Cr/Ac group (Table 2).

**Table 2:** Effect of crocin ± acrolein on oxidative stress and BALF inflammatory markers in different studied groups.

	Control group		Cr group		Ac group (n10)	Oral-Cr/Ac group (n10)	ip-Cr/Ac group (n10)	*p* value
	Oral-saline (n 5)	ip-saline (n 5)	Oral-Cr (n 5)	ip-Cr (n 5)				
**Oxidative stress markers**								
ROS (U/mg)	106.81 ± 2.30	106.86 ± 2.30	107.39 ± 2.32	107.61 ± 2.32	154.83 ± 3.26^b^	145.51 ± 3.60^bc^	109.79 ± 2.31^adf^	< 0.001
MDA (nmol/mg)	10.31 ± 0.22	10.32 ± 0.22	10.37 ± 0.22	10.39 ± 0.22	20.87 ± 1.24^b^	18.04 ± 1.07^bc^	11.39 ± 0.67^adf^	< 0.001
GSH (nmol/mg)	0.56 ± 0.01	0.56 ± 0.01	0.57 ± 0.01	0.56 ± 0.01	0.23 ± 0.01^b^	0.29 ± 0.01^bc^	0.56 ± 0.01^adf^	< 0.001
PCO (nmol/mg)	13.75 ± 0.30	13.76 ± 0.30	13.83 ± 0.30	13.86 ± 0.30	21.18 ± 0.44^b^	19.27 ± 0.40^bc^	14.28 ± 0.30^adf^	< 0.001
8-OHdG (ng/mL)	9.29 ± 0.20	9.30 ± 0.20	9.34 ± 0.20	9.36 ± 0.20	10.75 ± 0.63^b^	10.11 ± 0.60^bc^	9.87 ± 0.58^adf^	< 0.001
**BALF inflammatory markers**								
TNF-α (pg/mL)	29.02 ± 0.63	29.03 ± 0.63	29.18 ± 0.63	29.24 ± 0.63	42.32 ± 2.51^b^	40.15 ± 2.83^bc^	31.28 ± 1.85^adf^	< 0.001
IL-6 (pg/mL)	75.13 ± 5.88	75.17 ± 5.88	75.55 ± 5.91	75.70 ± 5.92	202.38 ± 4.26^b^	196.28 ± 4.13^bc^	79.05 ± 1.66^adf^	< 0.001
MIP-2 (pg/mL)	221.12 ± 12.64	221.23 ± 12.65	222.33 ± 12.71	222.78 ± 12.74	345.05 ± 7.27^b^	338.48 ± 7.13^bc^	227.47 ± 4.79^adf^	< 0.001

One-way ANOVA, and Tukey HSD Post-hoc Test, *p* > 0.05: no significant differences, *p* < 0.05: significant differences.

*BALF* Bronchoalveolar lavage fluid, *ROS* Reactive oxygen species, *MDA* Malondialdehyde, *GSH* Reduced glutathione, *PCO* Protein carbonyl, *8-OHdG* 8-Hydroxy-2′-deoxyguanosine, *TNF-α*: Tumor necrosis factor, *IL-6* Interleukin-6, *MIP-2* Macrophage inflammatory protein.

^a^*p* < 0.05 versus oral-saline group.

^b^*p* < 0.001 significant versus oral-saline group.

^c^*p* < 0.05 versus Ac group.

^d^*p* < 0.001 significant versus Ac group.

^e^*p* < 0.05 versus oral-Cr/Ac group.

^f^*p* < 0.001 significant versus oral-Cr/Ac group.

### Effect of acrolein ± crocin on BALF analysis

Results of TNF-α, IL-6, and MIP-2 demonstrated a significant elevation (*p* < 0.001) in Ac treated group when compared to the oral-saline group. In the group administered oral-Cr and Ac, results indicated a significant reduction (*p* < 0.05) in TNF-α, IL-6, and MIP-2 levels when compared to Ac-treated group, while a significant elevation (*p* < 0.001) was detected in comparison with the oral-saline group. In ip-Cr/Ac combined group, results revealed a significant reduction (*p* < 0.001) in the inflammatory parameters compared to Ac group and a significant elevation (*p* < 0.05) when compared to oral-saline group. A significant reduction (*p* < 0.001) in TNF-α, IL-6, and MIP-2 was detected in ip-Cr/Ac when compared to oral-Cr/Ac group (Table 2).

### Effect of acrolein ± crocin on histopathological lung examination

**Figure 1:**
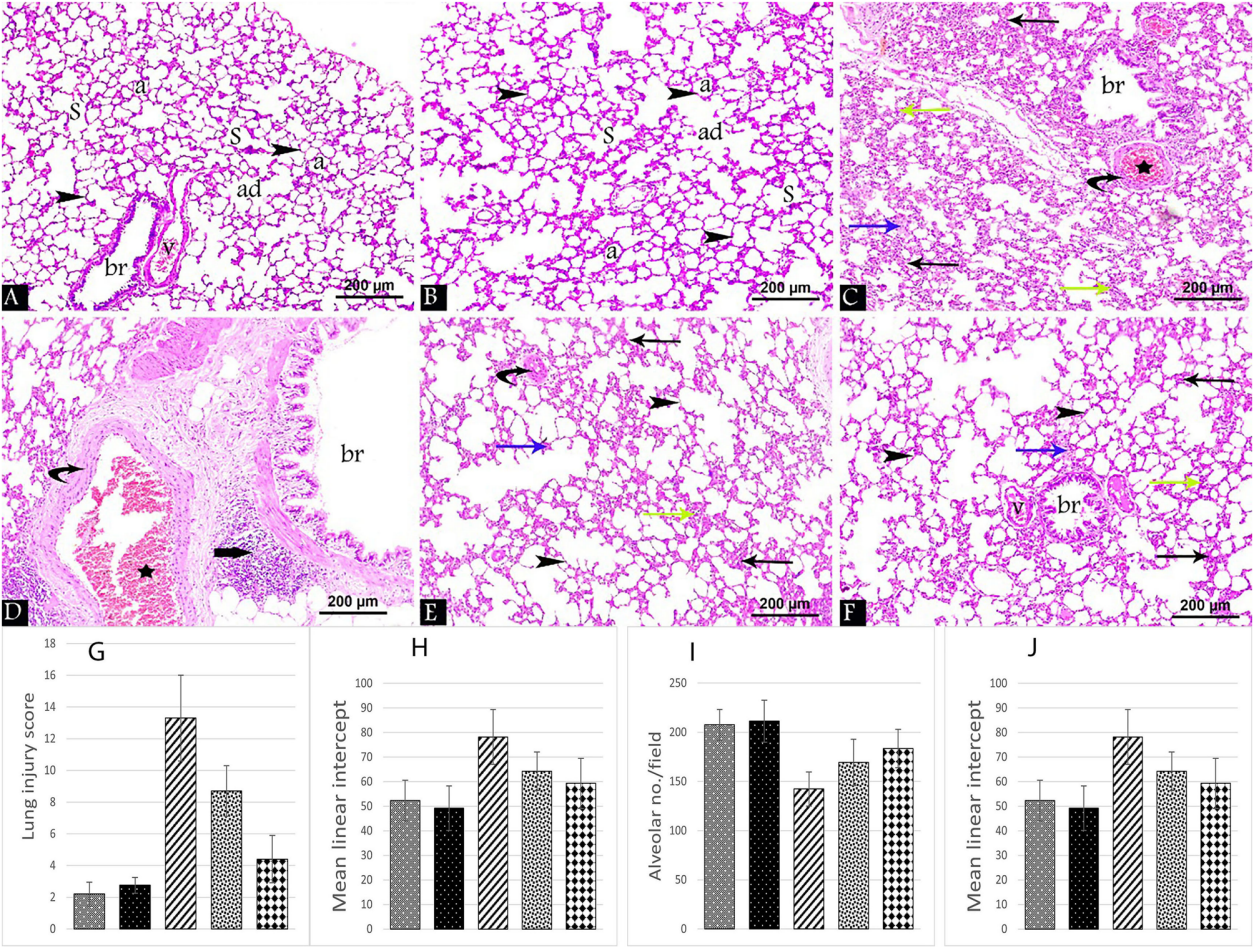
H&E-stained lung tissue sections at 400 × magnifications from all the groups showing alveolar ducts (ad), alveolar sacs (S) and alveoli (a) separated by thin interalveolar septa (arrowhead), normal respiratory bronchioles (br) accompanied by normal blood vessel (v) in control (**A**) and Cr groups (**B**). Ac group (**C**) & (**D**) shows thick interalveolar septa (black arrow), areas of perivascular and peri bronchial infiltration (thick arrow), interstitial exudate (blue arrow), interstitial infiltration (green arrow) and dilated congested (star) fibrosed (curved arrow) blood vessels. oral-Cr/Ac group (**E**) versus ip-Cr/Ac (**F**) showed that areas of thick interalveolar septa (black arrow), interstitial infiltration (green arrow) and exudate (blue arrow) are more prominent. Chart shows morphometrical analysis to lung injury score (**G**), radial alveolar count (**H**), mean linear intercept (**I**) and alveolar no./field (**J**) in all the groups.

H&E-stained lung tissue sections from the control and Cr groups displayed the classic histological structure that appeared as normal sized alveolar ducts, alveolar sacs, and alveoli separated by thin interalveolar septa. Respiratory bronchioles were lined with intact epithelium and accompanied by normal blood vessels (Fig. 1A,B). However, in Ac treated group disturbance of the normal histological structure was noticed with thickening of the interalveolar septa. Areas of perivascular and peri bronchial infiltration, interstitial exudate, infiltration, and dilated congested fibrosed blood vessels were also detected (Fig. 1C,D). Treatment with crocin in oral-Cr/Ac and ip-Cr/Ac groups showed improvement in the lung architecture which was more evident in ip-Cr/Ac group (Fig. 1F) compared to oral-Cr/Ac group (Fig. 1E). Thin interalveolar septa with few areas of thick septa were noticed in ip-Cr/Ac group. Meanwhile, the reverse was evident in the oral-Cr/Ac group where some of them were ruptured resulting in formation of large irregular air spaces. The interstitial infiltration, exudate, and dilated congested fibrosed blood vessels were less in ip-Cr/Ac, compared to the oral group.

**Figure 2:**
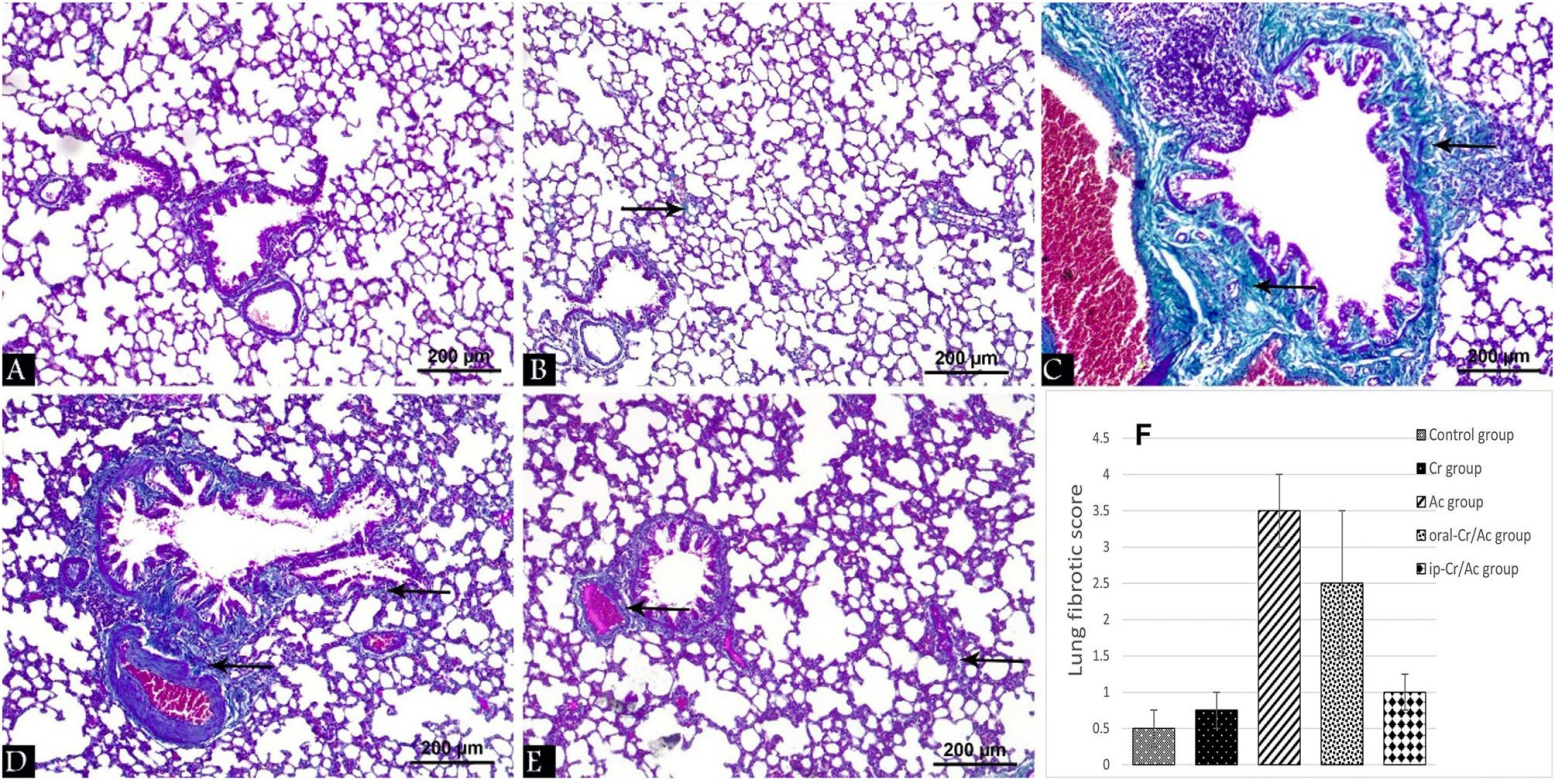
Masson’s trichrome stained lung tissue sections at 400 × magnification from all the groups showing collagen fibers (arrow). Control group (**A**), Cr group (**B**), Ac group (**C**), oral-Cr/Ac group (**D**), ip-Cr/Ac group (**E**). Chart shows morphometrical analysis to lung fibrotic score (**F**).

Masson’s trichrome stained lung tissue sections in the control and Cr groups revealed normal deposition of collagen fibers (Fig. 2A,B), whereas markedly increased collagen fibers were deposited in the interalveolar spaces and around bronchi and blood vessels in Ac group (Fig. 2C). A mild increase in the deposited collagen fibers was seen in ip-Cr/Ac group compared to the moderately increased amount in oral-Cr/Ac group (Fig. 2E,D) respectively.

### Effect of acrolein ± crocin on immunohistochemical apoptotic markers

**Figure 3:**
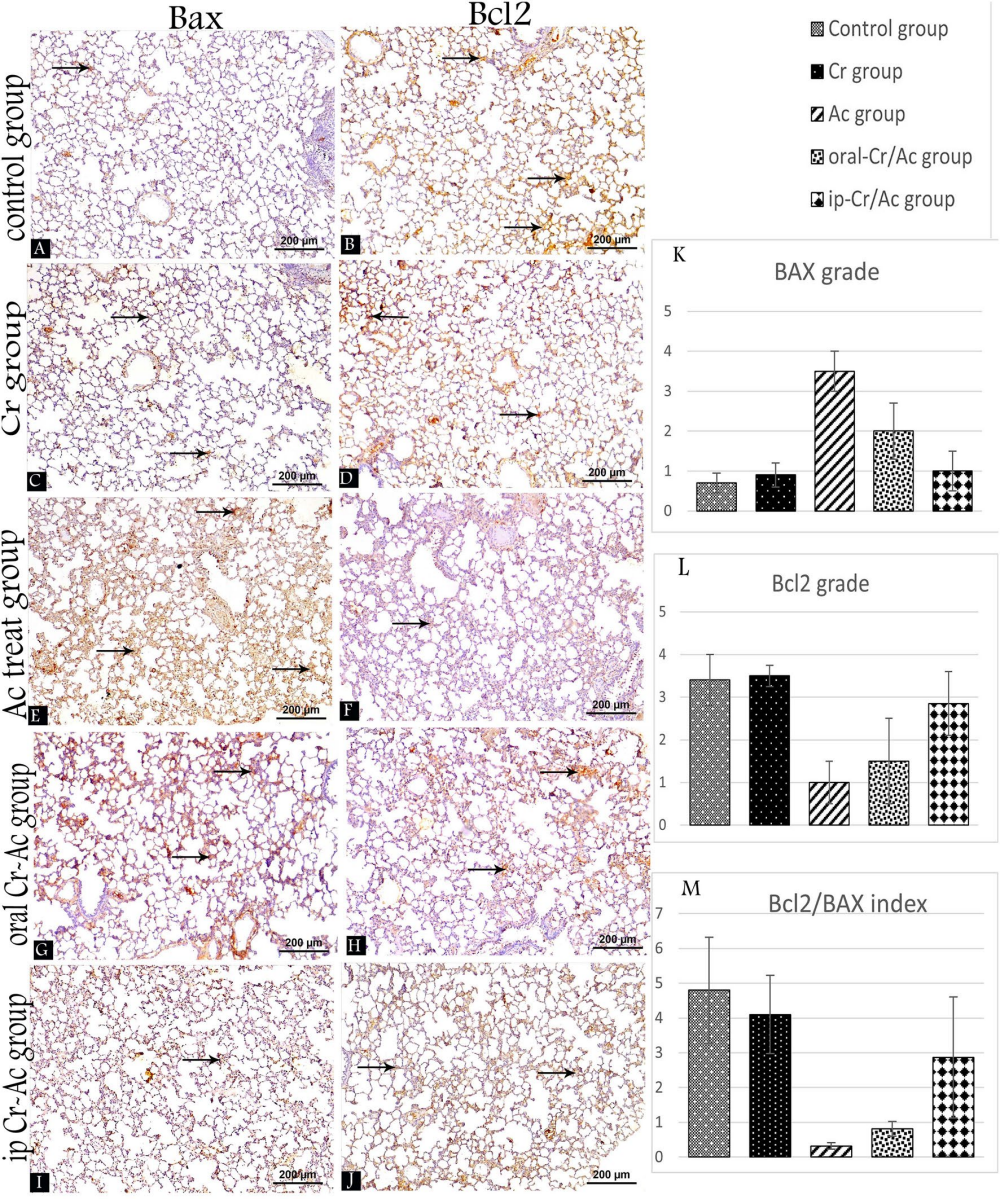
Bax and Bcl2 immuno-stained lung tissue sections at 400 × magnification from all the groups showing the degree of immunoexpression (arrow). Control group (**A**,**B**), Cr group (**C**,**D**), Ac group (**E**,**F**), oral-Cr/Ac group (**G**,**H**), ip-Cr/Ac group (**I**,**J**). Chart shows morphometrical analysis to Bcl2/Bax index (**M**) and grading of anti-BAX (**K**) and anti-Bcl2 (**L**).

To determine which route of Cr. administration can enhance more amelioration on Ac induced lung apoptosis, we studied Bcl-2 and Bax immunoexpression. The results showed that administration of Ac increased Bax expression and decreased Bcl-2 expression (Fig. 3E,F). In contrast, increased Bcl-2 expression, and decreased expression of Bax were observed in control and Cr. Groups (Fig. 3A–D). Moderate Bax expression was evident in oral-Cr/Ac group compared to the mild expression in ip-Cr/Ac group (Fig. 3G,I) while a moderate expression of Bcl2 was detected in ip-Cr/Ac group compared to the mild expression in the oral group (Fig. 3J,H) respectively.

### Histopathological and immunohistochemical image morphometrical analysis

H&E image morphometrical analysis showed a significant increase in lung injury score (*p* < 0.001) and mean linear intercept (*p* < 0.001), as well as a significant decrease in radial alveolar count (*p* < 0.001) and alveolar no./field (*p* < 0.001) in Ac group when compared to the control group.

In the group administered oral-Cr and Ac, results indicated a significant increase in lung injury score (*p* < 0.001), and a significant decrease in radial alveolar count (*p* < 0.001) and alveolar no./field (*p* < 0.001) when compared to the control group. When compared with Ac group, oral-Cr/Ac group revealed a significant reduction in lung injury score (*p* < 0.001) and mean linear intercept (*p* < 0.05), besides significant increase in radial alveolar count (*p* < 0.001) and alveolar no./field (*p* < 0.05). In ip-Cr/Ac group, all parameters showed significance (*p* < 0.001) improvement versus Ac group. When compared with oral-Cr/Ac group, lung injury score showed significant improvement (*p* < 0.001) (Table 3, Fig. 1G–J).

**Table 3:** Effect of crocin ± acrolein on histological and immunohistochemical morphometrical parameters of lung tissue in different studied groups.

	Control group (n10)	Cr group (n10)	Ac group (n10)	Oral-Cr/Ac group (n10)	ip-Cr/Ac group (n10)	*p* value
**H&E morphometry**						
Lung injury score	2.2 ± 0.75	2.75 ± 0.5	13.3 ± 2.7^b^	8.7 ± 1.6^bd^	4.4 ± 1.5^adf^	< 0.001
Radial alveolar count	39.4 ± 7.2	41.2 ± 5.4	19.4 ± 4.2^b^	27.3 ± 5.4^bc^	30.2 ± 4.3^ad^	< 0.001
Alveolar no./field	207.6 ± 15.6	211.3 ± 21.4	142.3 ± 17.3^b^	169.3 ± 23.5^bc^	183.4 ± 19.6^d^	< 0.001
Mean linear intercept	52.3 ± 8.3	49.2 ± 9.1	78.2 ± 11.2^b^	64.2 ± 7.8^c^	59.3 ± 10.2^d^	< 0.001
**Masson’s trichrome morphometry**						
Lung fibrotic score	0.5 ± 0.25	0.75 ± 0.25	3.5 ± 0.5^b^	2.5 ± 1^bc^	1 ± 0.25^df^	< 0.001
**Immunohistochemical morphometry**						
Anti-BAX grade	0.7 ± 0.25	0.9 ± 0.3	3.5 ± 0.5^b^	2 ± 0.7^ad^	1 ± 0.5^df^	< 0.001
Anti-Bcl2 grade	3.4 ± 0.6	3.5 ± 0.25	1 ± 0.5^b^	1.5 ± 1^b^	2.85 ± 0.75^df^	< 0.001
Bcl2/BAX index	4.8 ± 1.52	4.1 ± 1.12	0.32 ± 0.09^b^	0.81 ± 0.21^b^	2.86 ± 1.74^bdf^	< 0.001

One-way ANOVA, and Tukey HSD Post-hoc Test, *p* > 0.05: no significant differences, *p* < 0.05: significant differences.

^a^*p* < 0.05 versus control group.

^b^*p* < 0.001 significant versus control group.

^c^*p* < 0.05 versus Ac group.

^d^*p* < 0.001 significant versus Ac group.

^e^*p* < 0.05 versus oral-Cr/Ac group.

^f^*p* < 0.001 significant versus oral-Cr/Ac group.

Regarding to Masson’s trichrome morphometrical analysis, Ac group showed significant increase in lung fibrotic score (*p* < 0.001) versus control group. In oral-Cr/Ac group, lung fibrotic score revealed significant difference (*p* < 0.001) versus control group and significant difference (*p* < 0.05) versus Ac group. While lung fibrotic score in ip-Cr/Ac group revealed significant difference (*p* < 0.001) versus both Ac and oral-Cr/Ac groups with no significant difference versus control group (Table 3, Fig. 2F).

Moreover, immunohistochemical Bax grade “apoptotic marker” in Ac group showed a significant increase (*p* < 0.001) versus control group. In oral-Cr/Ac group, Bax grade revealed a significant increase (*p* < 0.05) versus control group and significant decrease (*p* < 0.001) versus Ac group. While Bax grade in ip-Cr/Ac group revealed a significant decrease (*p* < 0.001) versus both Ac and oral-Cr/Ac groups with no significant difference versus control group.

Moreover, immunohistochemical anti-Bcl2 grade “anti-apoptotic markers” in Ac group showed significant decrease (*p* < 0.001) versus control group. In oral-Cr/Ac group, anti-Bcl2 grade revealed a significant decrease (*p* < 0.001) versus control group. While anti-Bcl2 grade in ip-Cr/Ac group revealed a significant increase versus both Ac (*p* < 0.001) and oral-Cr/Ac (*p* < 0.001) groups with no significant difference versus control group. Moreover, despite that Bcl2/BAX index in Ac, oral and ip-Cr/Ac groups showed significant decrease (*p* < 0.001) versus control group, Bcl2/Bax index in ip-Cr/Ac group revealed also a significant increase (*p* < 0.001) versus Ac group and oral-Cr/Ac group (Table 3, Fig. 3K–M).

## Discussion

Results of the anthropometric measures in Ac treated group revealed a significant decrease in body weight, a significant elevation in wet lung weight, and a significant increase in wet/dry lung weight (W/D%) which are indicative of lung edema. These findings were in line with Lu et al.^1^ who reported increased lung wet-to-dry weight ratio and loss of body weight in Ac treated mice. Similarly, Jang et al.^26^ and Gao et al.^27^ stated that inhalation of Ac had caused pulmonary edema in animals and considered the W/D ratio as an indicator of lung edema.

In current work, the biochemical results revealed a significant elevation in oxidative stress markers (ROS, MDA, PCO, 8-OHdG) and reduction in GSH content in lungs of Ac-treated group compared to the oral-saline group. Variable mechanisms have been suggested to explain Ac mediated cytotoxicity, including oxidative stress, DNA damage, protein adduction, inflammation, cell membrane disruption, mitochondrial dysfunction, and endoplasmic reticulum stress^28^. Ac exhibits extreme reactivity towards biological nucleophiles like cellular thiols, cysteine, histidine, and lysine residues of proteins, as well as the nucleophilic sites of DNA^29^.

Adduction to macromolecules is the key mechanism of Ac-induced toxicity which initiates the cycle of oxidative stress, inflammation, tissue damage, cell death, and necrosis in respiratory tract^30^. Presence of different thiols like GSH and N-acetylcysteine provides a relative protection against Ac-induced cytotoxicity in lung. However, prolonged Ac adduction to thiols results in a concentration-dependent depletion of GSH in the respiratory mucosa and impairs redox homeostasis^30^. Also, Ac-induced ROS can disrupt nuclear factor erythroid 2-related factor 2 (Nrf2)-glutamate cysteine ligase (GCLc)-GSH pathway, resulting in reduced GSH expression and levels^8^.

Generation of ROS is enhanced by GSH depletion, formation of superoxide radicals as a byproduct of Ac metabolism by aldehyde dehydrogenase or xanthine oxidase enzymes, and inhibition of endogenous antioxidants^31^. Excess ROS react with polyunsaturated fatty acids in cell membranes resulting in lipid peroxidation and increased MDA levels^30^. In addition to ROS generation and lipid peroxidation, Ac can covalently bind to side-chain amine groups (lysine, arginine, proline, or histidine) of proteins resulting in formation of protein carbonyls which represent reliable markers for ROS mediated protein damage^32^. Also, Ac via direct adduction to DNA, can cause DNA damage and impair DNA repair. Besides, Ac via ROS can indirectly react with DNA resulting in formation of 8-OHdG, the most reliable marker for oxidative stress mediated DNA damage^33^.

Parallel to our results, in male C57BL/6 mice subjected to acute Ac inhalation (10 ppm for 12 h), oxidative stress markers (MDA, GSSG: GSH ratio, 8-OHdG, and PCO) has markedly increased^24^. Also, Ac at a dose of 2.5 mg/kg/day for 8 weeks, has resulted in increased MDA level, reduced GSH, and depleted thiols in livers of Wister rats^29^. Similarly, mice orally gavaged with Ac (7.5 mg/kg/day, gavage) for 3 weeks has demonstrated high MDA levels, low GSH and CAT levels, and excess ROS in cardiomyocytes of treated animals^34^. Luo et al.^35^ has also reported increased ROS generation and MDA levels in spinal cord of Ac treated rats, in a time and dose dependent manner. Furthermore, Ac exposure at a dose > 50 um over 24 in ARPR19 cells, has diminished GSH, total antioxidant capacity, SOD, and GPx levels, and increased oxidants and PCO levels^8^. Also, accumulation of PCOs within the myocardium has been reported in mice injected with Ac (1 mg/kg/day intravenous)^36^.

In the present study, combined administration of Cr and Ac resulted in a relative mitigation of oxidative stress biomarkers (ROS, MDA, PCO, GSH, and 8-OHdG) in oral-Cr/Ac group compared to the oral-saline group, while nearly normal parameters were shown in the combined group administered ip-Cr along with Ac, which indicates a more potent effect of ip-Cr over the orally administered Cr.

Crocin is a naturally derived antioxidant with a direct antioxidant effect. The antioxidant capacity of Cr has been attributed to its ability to inhibit lipid peroxidation, protein carbonylation, and DNA damage in cells^37^. Moreover, Cr represents an efficient free radical scavenger owing to the hydroxyl and glucose moieties in its molecular structure^21^. Also, Cr improves total thiols and increases GSH pool via modulation of ROS-Nrf2-GCLc-GSH pathway^13^. Besides, increased GSH pool and related enzymes (such as GSH peroxidase, GSH reductase, and GSH -S-transferase), enable Cr to suppress genotoxicity. Consistently, in previous study, Cr administration has successfully reduced 8-OHdG levels and downregulated p53 gene, the guardian of genome induced by DNA damage and ROS^38^.

Following oral administration, crocin has revealed poor absorbability and limited oral bioavailability (0.3–0.6‰). Interestingly, crocin can’t permeate into the bloodstream as the prototype, but it undergoes rapid hydrolysis into crocetin in the gastrointestinal tract. Hereafter, crocetin finds its access to the plasma via simple diffusion through the intestinal wall, resulting in 56 to 81-fold higher plasma crocetin exposure than crocin^15,18^. Crocetin extensively distributes to the liver where it is partially metabolized by glucuronidation into mono and diglucuronides conjugates^39^.

There is no evidence suggesting crocetin accumulation in plasma, after neither single dose nor repeated doses administration of crocin, which indicates rapid elimination and short plasma half-life of crocetin^14^. Furthermore, nearly 80% of oral crocin is directly eliminated from the body without metabolic absorption, while 20% of dose is eliminated in stool as intact crocetin, monoglucuronide conjugates, and diglucuronide conjugates^14^.

Actually, oral route of crocin administration seems to be the only source for crocetin production via intestinal hydrolysis. On the other hand, no crocetin has been detected in plasma or fecal samples of rats administered crocin parenterally, where solely crocin has been found in circulation^17^. Unlike the oral route, crocin exhibits higher absorbability and bioavailability with parenteral administration, and distributes rapidly and extensively to heart, lung, kidney and spleen owing to the high-water solubility^17,18^.

Both crocin and crocetin possess anti-inflammatory, antioxidant, and anti-apoptotic effects. The antioxidation and anti-inflammation contribute mainly to the therapeutic role of crocin in different diseases. Meanwhile, the anti-apoptotic effect represents the key mechanism in minimizing cell damage associated with pathological disorders^40^. Noteworthy, the limited pharmacokinetic studies necessitate more research for better understanding of the precise pharmacokinetics of different routes of crocin administration.

The antioxidant effect of ip-Cr has been previously reported in many studies; Cr (50 mg/kg, ip, 3 times/week, and 50 mg/kg, ip once/day for 2 months) has significantly decreased cigarette smoking (CS)-induced ROS production and increased GSH content^13^. Similarly, Cr has efficiently restored GSH pool in liver tissue with beryllium chloride^21^ and induced effective protection against experimentally induced ulcerative colitis in rats^41^. In same line, Cr administration has improved the oxidant/antioxidant balance in kidney tissues of periodontitis model rats^42^.

Reduced 8-OHdG in Cr/Ac combined groups was in line with the anti-genotoxic effect reported by Akbari and Mard^43^ in livers of Cr/cisplatin treated rats. Similarly, concomitant administration of Cr in a rotenone model of Parkinsonism could diminish 8-OHdG in serum via potent antioxidant effect^38^.

In lung, the oxidative stress mediated cell damage induced by Ac initiates tissue inflammation. In turn, inflammation leads to more cell damage, tissue destruction, and remodeling in respiratory tract, especially in tracheobronchial and pulmonary regions. On the other hand, inflammation enhances production of more ROS which exacerbate the imbalance of redox system in respiratory tract^30^.

In the present study, results of BALF analysis demonstrated increased levels of inflammatory mediators (TNF-α, Il-6, MIP-2) in Ac-treated group, that were partially ameliorated by oral-Cr co-administration. On the other hand, a nearly full protection was achieved in the ip-Cr/Ac combined group, indicating more efficient anti-inflammatory effect of the ip-Cr over the orally administered crocin. In support of our results, Ac (3 ppm for 14 and 28 days) in exposure chambers has resulted in elevated TNF-α and CINC-1 (analogue of IL-8 in rats) levels in the BALF and lung homogenate^44^. Similarly, mice exposed to Ac for 4 days has exhibited inflammatory response as indicated by increased inflammatory markers in bronchial fluid^45^. Also, increased TNF-α, and IL-1β in lung of mice exposed to Ac (10 ppm for 12 h) inhalation has been reported by Kim et al.^24^. In the present study, Cr co-administration with Ac has proved anti-inflammatory effect in lung. This can be ascribed to the potent antioxidant effect of Cr which suppresses the expression and productions of inflammatory cytokines like TNF-α, IL-6, and IL-1β^46^. Consistent with our results, Cr has reduced gene expression and levels of IL-6 and TNF-α, prevented inflammatory cells recruitment, and improved lung injury in previous studies^13,46,47^.

Apoptosis has been considered as an important mechanism for developing acute respiratory distress syndrome^48^. Also, determining the integrity of lung epithelium may be strongly correlated to the balance between pro-apoptotic and anti-apoptotic factors^49^. Lastly, it has been proved that of Bcl-2/Bax ratio is an important index indicating the severity of apoptosis^50^. Thus, the immunohistochemical study to detect apoptosis was performed in the current study.

Results showed marked apoptosis in Ac group, but co-administration of Cr downregulated expression of Bax and upregulated Bcl-2 expression, which decreased the rate of apoptosis in the lung tissue that was more evident in ip-Cr/Ac group. These findings were supported by Kim et al.^24^ who found that Ac had activated different apoptotic markers that were inhibited by naringin.

The previous elevated oxidative, inflammatory, and apoptotic markers were further supported by the histopathological findings. In the current study histopathological results revealed thickening of the interalveolar septa, increased MLI, perivascular and peri-bronchial infiltration, interstitial exudate and dilated congested fibrosed blood vessels in Ac treated group. This was in harmony with Wang et al.^44^ and Park et al.^51^ who observed severe destruction, thickened epithelium, peri-bronchial inflammatory cell infiltration, obstruction of the lumen by cell debris, and mucus and irregular pattern of alveoli in Ac treated mice and rats. On the other hand, lung tissue damage was reversed by co-administration of Cr particularly in the group administered Cr intraperitoneally. This result goes hand in hand with Dianat et al.^13^ who observed improvement in the lung tissue structure and decrease of MLI in crocin co-treated rats compared to cigarette smoke treated group.

Because fibrosis is recognized as a diagnostic indicator of lung injury, Masson's Trichrome staining was done to assess the score of lung fibrosis that showed high significant increase in Ac group compared to the other groups. These results cope with Kim et al.^24^ who found that fibrosis score in acrolein treated mice was nearly double the value recorded in control groups. Pulmonary fibrosis is well-known to provoke irreversible structural changes and is considered as one of the main causes of death via initiating apoptosis^52,53^. The resultant destruction and remodeling of lung tissues impair lung elasticity and compliance with enlargement of airspaces, producing the pathognomonic changes of COPD^30^.

Finally, the histopathological and immunohistochemical results were in accordance with our aforementioned results, indicating a more potent effect of ip-Cr over the orally-Cr, as previously discussed.

## Conclusion

In conclusion, intraperitoneal administration of crocin exhibited a more protective effect on acrolein-induced lung toxicity than oral crocin, mostly via antioxidant, anti-inflammatory, and anti-apoptotic mechanisms.

## Data Availability

The datasets generated and analyzed during the current study are available from the corresponding author on reasonable request.
